# Lessons Learned From an Analysis of the Emergency Medical Services’ COVID-19 Drive-Through Testing Facilities in Israel

**DOI:** 10.1017/dmp.2021.50

**Published:** 2021-02-16

**Authors:** Itay Zmora, Evan Avraham Alpert, Uri Shacham, Nisim Mishraki, Eli Jaffe

**Affiliations:** 1 Faculty of Medicine, Hebrew University of Jerusalem, Israel; 2 Department of Emergency Medicine, Shaare Zedek Medical Center, Jerusalem, Israel; 3 Magen David Adom, Tel Aviv, Israel; 4 Ariel University, Ariel, Israel; 5 Department of Emergency Medicine, Ben Gurion University of the Negev, Beer Sheva, Israel

**Keywords:** pandemics, COVID-19, COVID-19 diagnostic testing, emergency medical services

## Abstract

One strategy for the containment of a pandemic is mass testing. Magen David Adom (MDA), the Israeli National Emergency Medical Services (EMS) Organization undertook this mission by operating a nationwide series of drive-through testing complexes. The objective of this study is to learn lessons from an analysis of these centers. Data from 198 stationary and mobile drive-through complexes from March 20, 2020, through October 17, 2020, were analyzed for temporal and geographic factors, and cost. Also, an operational improvement program was implemented and analyzed. A total of 931,074 patients were sampled in the MDA drive-through system: 46.9% in stationary complexes, and 53.1% in mobile complexes. The optimized cost per patient of home testing was estimated at 74.5 USD compared with 6.55 USD in the drive-through centers. An operational improvement program lowered the total sampling time from 128 s/patient to 98 s and decreased the total cost per patient from 6.55 USD to 6.27 USD. The EMS led drive-through complexes were cost-effective and efficient in performing large numbers of viral tests, especially when compared with home testing. Established concepts in clinical operations should be implemented to increase the number of persons that can be tested and decrease cost.

The coronavirus disease 2019 (COVID-19) pandemic is a major challenge for health-care systems worldwide. According to the World Health Organization, as of November 1, 2020, there were more than 45.5 million confirmed cases of COVID-19 and nearly 1.2 million deaths.^1^ The key to managing the crisis is containment or at least mitigation of the pandemic.^2^ One strategy to achieve this requires mass testing.^3^ Options include examinations in the home, community clinics, and emergency departments.^4-6^ An additional option is a drive-through system.^7^ The concept itself has been described in the past in an attempt to prepare for a potential influenza epidemic.^5^ The advantages and disadvantages of the drive-through testing facility have been detailed by Choi et al.^4^ The benefits include high efficiency, privacy, and reduced risk of staff infection. The disadvantages include construction costs, relevancy only to car owners, exposure of the staff to weather conditions, and crew fatigue. Several studies described their experience with the drive-through system during the COVID-19 pandemic.^8-16^ While all of them found it efficient and safe, most have short term follow-up and relatively small numbers of patients. There is also emerging research on other uses of drive-through testing centers during COVID-19, such as transcutaneous bilirubin screening for neonatal jaundice,^17^ prenatal care,^18^ and as a substitute for anticoagulation clinics.^19^

In Israel, as of November 1, 2020, a total of 314,535 patients have detected positive with COVID-19 and 2541 died.^20^ Since the beginning of the pandemic, the Israeli Ministry of Health (MOH), a government agency, charged Magen David Adom (MDA), the Israeli National Emergency Medical Services Organization with taking an active response to contain the virus. MDA carried out this mission on multiple levels including operating a dedicated COVID-19 call center,^21^ ambulance transport, and testing. Initially, paramedics were dispatched to the homes of suspected COVID-19 patients to perform nasal swabs. The MOH requested MDA to increase the number of tests which led to the establishment of drive-through centers.

## Methods

The policy of the MOH was to conduct mass testing while preventing patients with disease from entering clinics and emergency departments to reduce the chance of infection of medical staff and other patients. At the beginning of the pandemic, the scope of testing was small. A concerned patient would call the MDA designated COVID-19 call center. If they met specific criteria including exposure to a verified patient or appropriate symptoms, they would be referred to a physician to decide on the need for testing.^21^ If testing was determined to be necessary, a paramedic was dispatched to the patient’s house to obtain samples. This approach resulted in a relatively small number of tests at a high cost. As the pandemic spread, the MOH directed MDA to significantly increase the number of tests.

To achieve this, MDA decided to set up drive-through testing complexes. Four stationary drive-through testing complexes (SDTTC) were established in the largest cities in Israel: Jerusalem, Tel Aviv, Haifa, and Beer Sheva. Also, 8 mobile drive-through testing complexes (MDTTC) were set up in remote or other high-volume areas. The SDTTC included several lanes, whereas the MDTTC usually consisted of only 1 route but could be expanded.

Patients were referred to the drive-through testing centers by contacting the national emergency response number 1-0-1. An automated reply directed anyone with suspicious symptoms to the dedicated MDA COVID-19 call center staffed 24 hr a day by trained personnel. By means of telephonic questioning, if there was epidemiologic exposure without symptoms, the patient was directed to stay in home isolation for 14 d. If there were epidemiologic exposure and symptoms of cough, difficulty breathing, and/or fever above 38°C, a request opened for COVID-19 testing. In the case of potential emergent complaints, an ambulance staff was dispatched to the patient’s home in full personal protective equipment (PPE). In all cases, this included a gown, gloves, an N-95 face mask, and a face shield.

Stable patients were asked if they own a vehicle and can reach a drive-through complex. If the answer was negative, a paramedic was dispatched to the patient’s house for sampling. If the answer was affirmative, a text message was sent with the option of selecting the date and location. They were also asked to upload personal identification or driver’s license with photo. Then the patient received another SMS with a QR code, which they presented on arrival at the test compound.

When a vehicle arrived at the complex, it was directed to 1 of the tracks, and the driver was instructed to close all windows and turn off the air conditioning. When the vehicle arrived at the sampling tent, each person was asked to present the QR code to a barcode scanner through a closed window. The vehicle advanced to the sampling position staffed by 2 workers: 1 with PPE performed the sampling, and the other verified identification labels on the specimens. The vehicle was directed to the exit lane, and the sampler put the specimen into a biohazard bag. The sampler changed gloves and washed hands between each case.

Data were obtained for a series of 4 SDTTC and 8 other MDTTC that were operated in over 194 locations during the period from March 20, 2020, through October 17, 2020. All data in the form of an Excel spreadsheet (Redmond, WA: Microsoft) were taken from the command and control system of MDA. The study was retrospective using de-identified data and involved no patient interventions. It received the official approval of the Scientific Committee of MDA, and a waiver for patient consent from the Institutional Review Board (Helsinki Committee) of the Shaare Zedek Medical Center (0098-20).

## Results

### Testing

**Figure 1.**
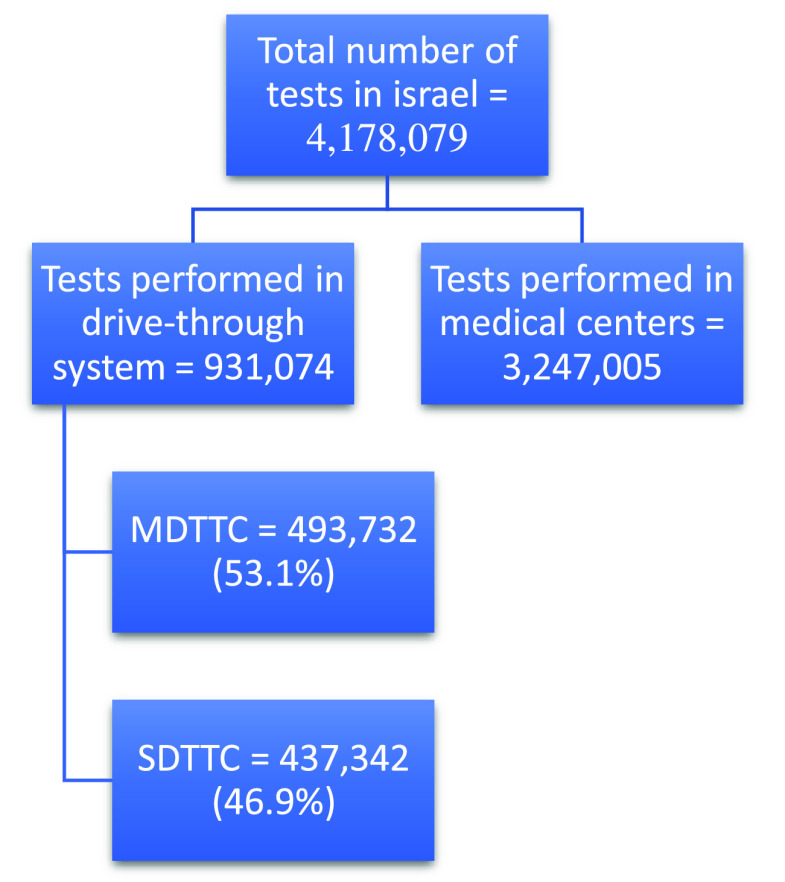
Number of tests performed during the study period. MDTTC, mobile drive-through testing complex; SDTTC, stationary drive-through testing complex.

**Figure 2.**
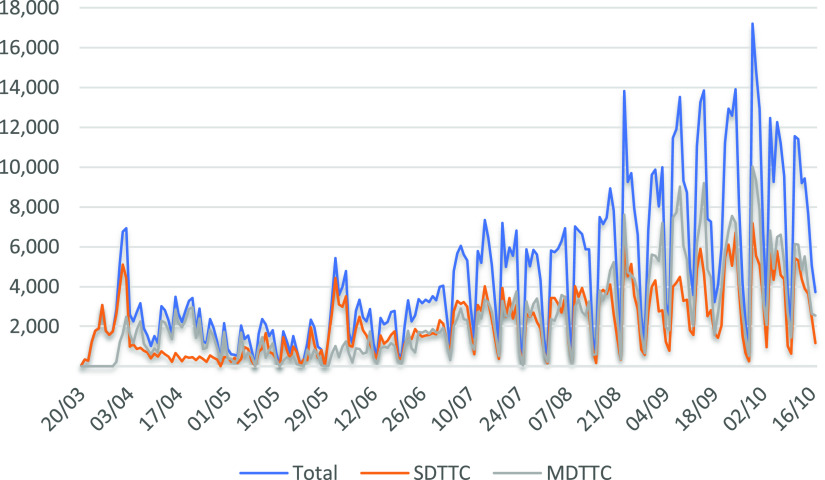
The total number of tests performed in all drive-through complexes throughout the study period. Total, the total of the stationary plus mobile drive-through complexes; SDTTC, stationary drive-through testing complexes; MDTTC, mobile drive-through testing complexes.

**Figure 3.**
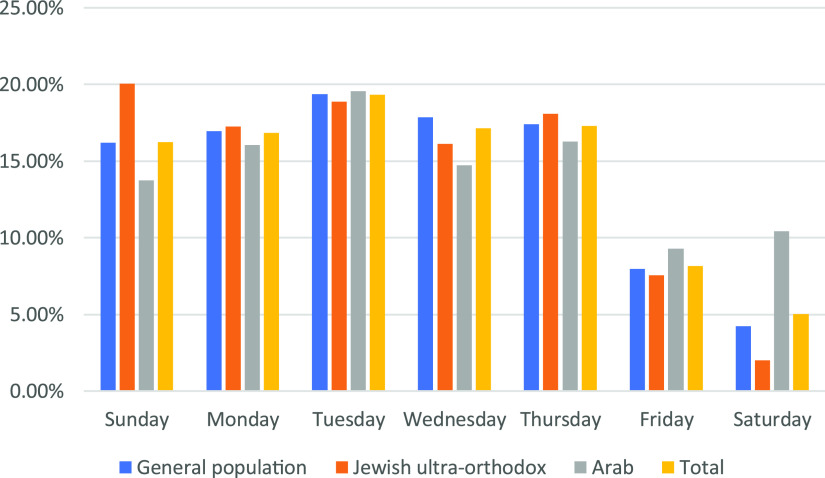
Percentage of samples by weekdays by ethnicity. General population, Jewish, not ultra-orthodox.

From March 20 through October 17, 2020, a total of 4,178,079 diagnostic tests were performed in Israel including from hospitals and community medical services. Of these, 931,074 patients were sampled in the MDA drive-through system; 437,342 in SDTTC, and 493,732 in 194 locations in MDTTC (Figure 1). From the beginning of April when the MDTTC opened, the number of tests conducted in them increased relative to the number of tests in the SDTTC (Figure 2). Throughout the week, most patients arrived Sunday through Thursday (Figure 3).

### Cost Analysis

Each paramedic who traveled to sample a patient at home required a driver, a vehicle, and a protective suit for each test. Each group of 4 teams was coordinated by 1 manager at the call center. Each sample took approximately 1 hr, including a trip to the home, donning full PPE, sampling, and removing the PPE. The cost per employee per hour was approximately 14 US dollars (USD); 2.25 employees per test equaled 31.5 USD. Each PPE cost approximately 35 USD. Vehicle and gasoline costs (administrative costs) were estimated at 8 USD/h. The total cost of performing each test along with vehicle expenses was approximately 74.5 USD.

In the drive-through complexes, initially, each lane was operated by 3 workers, 2 of whom wore full PPE (the workers in the sampling position). The crew members changed their PPE every 2 hr, which meant a total of 1 suit was used every hour. The total hourly cost of 3 crew members was 42 USD along with the hourly cost of 1 PPE of 35 USD, totaling 77 USD/h. According to MDA, 20 patients on average (although in urban areas there were more with less in the periphery) could realistically be examined per hour in each lane resulting in the cost of 3.85 USD per patient. The fixed cost of setting up a 3-lane SDTTC was 30,000 USD, whereas a 1-lane MDTTC was 10,000 USD. The fixed cost for 4 SDTTC was 120,000 USD and 8 MDTTC was 80,000 USD, which totaled 200,000 USD for 20 lanes. During April, a total of 74,141 patients were sampled, which meant an administrative cost (average fixed cost) of 2.7 USD per patient. The total cost per patient by means of a drive-through station was 6.55 USD per patient versus 74.5 USD for a home visit (Table 1).

**Table 1.** Cost of home testing versus drive-through testing per patient^a^

	Home testing 1 patient/h			Drive-through testing 20 patients/h			Drive-through testing after improvement process 26 patients/h		
	Personnel^b^	PPE	Administrative costs ^c^	Personnel	PPE	Administrative costs ^d^	Personnel	PPE	Administrative costs ^d^
Units/h	2.25	1	-	3	2	-	4	3	-
Units per patient	2.25	1	-	0.15	0.05	-	0.15	0.06	-
Unit cost (USD)	14	35	8	14	35	2.7	14	35	2.07
Units per patient x unit cost (USD)	31.5	35	8	2.1	1.75	2.7	2.1	2.1	2.07
Overall cost/patient (USD)	**74.5**			**6.55**			**6.27**		

a Home testing assuming 1 patient/h.

b Includes paramedic, driver, and manager.

c Includes estimated cost of vehicle and gasoline.

d Includes fixed costs of setting up the stations.

Abbreviation: PPE, personal protective equipment.

### Operational Improvement Process

MDA incorporates volunteers on all levels, including support staff. As work began on the drive-through complexes, an industrial engineering consultant volunteered to explore the possibility of streamlining the process. Initially, 1 lane was examined and times were measured for the arrival of the vehicle at the entrance of the complex to the test position, the sampling time for all patients in the vehicle, and the individual sampling time.

Eleven cars were tested. The average arrival time from the waiting position to the test position was 66 s, the average sampling time was 109 s, and the average total time for each patient was 128 s (Table 2). The cause of any delay in each case was recorded. In 7 cases, there was a delay in the replacement of gloves, in 1 case there was a problem with the brightness of the smartphone screen such that the QR code could not be read, in 2 cases there was a combined problem of changing gloves and the brightness of the smartphone screen, and in 1 case the problem was not documented.

**Table 2.** Times measured^a^

Vehicle no.	No. of patients in car		Time for vehicle arrival (s)		Total sampling time (s)		Total sampling time per patient (s)		Total time per vehicle (s)		Total time per patient (s)	
	A	B	A	B	A	B	A	B	A	B	A	B
1	1	3	11	162	117	340	117	113.3	128	502	128	167
2	1	2	10	350	75	150	75	75	85	500	85	250
3	3	1	68	20	301	40	100.3	40	369	60	123	60
4	2	1	22	15	90	66	45	66	112	81	56	81
5	2	1	80	40	76	39	38	39	156	79	78	79
6	1	1	207	30	122	32	122	32	329	62	329	62
7	1	1	23	20	63	30	63	30	86	50	86	50
8	1	1	74	18	53	29	53	29	127	47	127	47
9	1	1	63	22	56	80	56	80	119	102	119	102
10	2	2	90	62	190	172	95	86	280	234	140	117
11	1	1	78	29	62	40	62	40	140	69	140	69
Average	1.5	1.4	66.0	69.8	109.5	92.5	75.12	57.3	175.5	162.4	**128.3**	**98.6**

a A, Times measured before improvement measures; B, Times measured after improvement measures.

To solve the glove replacement delay problem, another worker was added in each test position so that after 1 sample while 1 worker replaces gloves, the other worker was already sampling the next vehicle. To solve the smartphone screen brightness problem, signs were posted at the entrance to each lane asking patients to maximize the screen brightness of the smartphone. Then the times were tested for 11 cars and the average time of arrival was 69.8 s, the average sampling time was 162.4 s, and the average time for each patient was 98.6 s (Table 2).

After improving the process, the average test time dropped from 128 s to 98 s, a 30-s improvement per person. Given 20 patients/h, 600 s can be saved each hour, adding 6 additional patients per lane each hour. Assuming that each lane works for 12 h/day, an additional 72 patients per lane per day and over 2000 additional patients per lane per month can be seen.

However, this change added another staff member with a cost of 14 USD/h plus PPE for every 2 h with a cost of 17.5 USD/h. The total cost of manpower increased from 42 to 56 USD/h and the cost of PPE/h rose from 35 to 52.5 USD, totaling 108.5 USD/h. Given that 26 patients could be checked every hour, the cost per patient would increase to 4.17 USD per patient compared with 3.85 USD before improving the process.

Postprocess improvement 30% more patients could be tested. Assuming that the cost of setting up the complexes will remain similar, the administrative cost per patient would decrease to 2.07 USD compared with 2.7 USD before the process improvement strategy. The total cost per patient after the process improvement is expected to be approximately 6.27 USD compared with 6.55 USD before the improvement.

## Discussion

One of the primary methods to contain and mitigate a pandemic is to identify and isolate the carriers through mass testing.^22^ Traditionally, patients are examined in medical clinics and hospitals. Due to the high COVID-19 infection rate, there is a significant concern that patients who visit these sites to be examined will themselves spread the infection.^23^ Solutions to this problem include home sampling and drive-through stations.^6,7^ Siegler and colleagues explored the willingness of persons experiencing symptoms to seek testing. They found that 71% of were willing to be tested at a drive-through complex compared with 60% willing to be tested at a clinic.^24^

This article demonstrates that a national EMS organization can build and operate drive-through testing centers on a large scale. During the study time, over 930,000 tests were performed out of a total population of 9.2 million.^25^ MDA in particular has extensive experience with mass casualty incidents, but more importantly, protocols and procedures in place to deal with disasters on a national level.

Other unique features included preregistration by means of a national call number, invitation by text message, and identification of the patient at the site by QR code by means of a closed window. These simple applications of existing technology facilitated distancing between the staff and the patient as opposed to other systems that had onsite registration.

Once MDTTC opened, there was a steady increase in the number of tests performed there compared with the SDTTC. This is probably due to convenience as they were closer to one’s home. Another possible reason is the desire of people to avoid crowded places in times of pandemics. It may be preferable to invest in setting up a large number of mobile complexes, each of which is relatively inexpensive compared with a small number of larger, more expensive centers. They are also easy to transport and set up as they consist of gazebo-like structures that can simply be folded and put into an empty ambulance. It also seems logical to place the MDTTC near where the outbreak is concentrated. The costs of the drive-through visits versus home findings are consistent with previous studies.^9,12,16,26^

Overall, fewer patients came for tests on Fridays and Saturdays, which are considered the weekend in Israel. Israel’s population consists of 74% who identify as Jewish and 21% Arabs. Israeli Arabs are mostly Muslim (83%), with Christian Arabs (9%), and Druze (8%).^27^ The Arab population is least tested on Fridays, which is the Islamic holy day of the week, and ultra-Orthodox Jews are least tested on Saturday, which is the traditional Jewish day of rest when driving is prohibited except for emergencies. It seems logical to operate the complexes in a manner that is appropriate for the population being tested. For example, shutting down or reducing manpower at times that are projected to see fewer visits, but expanding services when one anticipates a large number of visits.

Home testing is significantly more expensive than testing at the drive-through complexes. Mark et al. compared hospital with home testing and found that those performed at home were cheaper. However, this team only tested approximately 80 patients over 2 wk.^6^ This study also shows the limited ability to sample large numbers of patients through home testing. Another benefit that Mark et al. noted was that out-of-hospital testing lowered the chance of infecting medical staff and other hospital visitors. As far as we know, no staff member who worked in MDA’s drive-through complexes became infected with COVID-19 during the study period.

It should be noted that the longer the complexes operate, the more the cost per patient decreases, but maintenance costs will be added and the costs should be recalculated.

Literature in the field of clinical operations exists for many high-volume medical facilities, including the emergency department, medical clinics, and operating room.^28-30^ MDA operates with a salaried staff as well as volunteers.^31^ Most of these volunteers are at the level of an emergency medical technician or paramedic; however, some help with the clinical operations on an ad hoc basis. Due to a large number of visits, MDA turned to an industrial engineering consultant who volunteered time to improve the drive-through testing process. The results show that the use of improvement processes can streamline work and lower costs. Other studies have also emphasized process improvement, such as arranging separate “slow” lanes for families with younger children or multiple children.^14^

## Limitations

This study has several limitations. The research period is relatively short and does not allow a long-term perspective that can affect both costs and capabilities to maintain such an array over time. There may be seasonal variations in cost such as heating or cooling. During the spring, summer, and early fall seasons when the testing took place, there was almost no rain. If this would be implemented in the winter then additional costs of heating and rain-proofing would need to be taken into consideration.

The quality improvement process is based on the ideal situation and doesn’t take into effect worker fatigue from repetitive routine actions in personal protective equipment over long periods during the stresses of a pandemic.^32,33^ It also does not consider the fact that there may be technical malfunctions with operating the lanes or that workers themselves over time may become sick, and so fewer lanes will be available for operation. It also requires validation within MDA and external validation by other facilities. The cost analysis is based on optimized operational capacity, but there were times such as when the pandemic wave was waning that throughput was not being maximized.

There is a known limitation to the sensitivity of the tests^34^ and a known swabbing technique that requires insertion through the nostril to the nasopharynx.^35^ There was no quality control procedure in this study. The tests were performed by laboratories that notified the MOH of the results, which then conveyed them to the patients. MDA was not aware of the test outcomes.

## Conclusions

Emergency medical services can build and operate drive-through complexes that offer a solution that is cost-effective, safe, and efficient in performing large numbers of viral tests, especially when compared with home testing. Mobile drive-through centers appear to be more effective than stationary complexes. Established concepts in clinical operations should be implemented to increase the numbers of persons that can be tested and decrease the costs. Simple use of existing technology should be incorporated, including preregistration by means of a phone triage system, invitation and instruction by text messaging, and patient identification by a QR reader through a closed window. Future studies should examine quality control, further process improvement, and the impact on pandemic containment and mitigation.

Author Contributions. Dr Zmora and Dr Alpert are equal first contributors.
